# Aggressive Natural Killer (NK)-Cell Leukemia Initially Presenting as Orbital Cellulitis: A Case Report

**DOI:** 10.7759/cureus.117536

**Published:** 2026-10-06

**Authors:** Mitsuhiko Koseki, Yuta Kitamura, Yusuke Kouchi, Sakurako Harada-Kagitani, Takayuki Baba

**Affiliations:** 1 Department of Ophthalmology and Visual Science, Chiba University Graduate School of Medicine, Chiba, JPN; 2 Department of Molecular Pathology, Chiba University Graduate School of Medicine, Chiba, JPN; 3 Department of Pathology, Chiba University Hospital, Chiba, JPN

**Keywords:** aggressive nk-cell leukemia, hemophagocytic syndrome (hs), idiopathic orbital inflammation, nk/t-cell lymphoma, orbital cellulitis mimic

## Abstract

We report a case of aggressive natural killer cell leukemia (ANKL) presenting as orbital cellulitis, highlighting diagnostic challenges for ophthalmologists. A 50-year-old Japanese man presented with progressive right eyelid swelling and was initially diagnosed with orbital cellulitis. Antibiotics were ineffective. Given the antibiotic-refractory orbital inflammation without sinusitis, corticosteroids were initiated but provided only transient improvement. After hospitalization with pulse methylprednisolone, the patient developed fever, thrombocytopenia, and hepatic dysfunction, with markedly elevated ferritin, soluble interleukin-2 receptor, and plasma Epstein-Barr virus (EBV) DNA, and features suggestive of hemophagocytic lymphohistiocytosis. Bone marrow and liver biopsies subsequently demonstrated a natural killer (NK)-cell neoplasm positive for CD56 and for EBV-encoded small RNA by in situ hybridization, with expression of T-cell intracellular antigen-1 and granzyme B in the liver. Leukemic bone marrow infiltration, concurrent sinusoidal infiltration of the liver without a mass lesion, the absence of nasal involvement, and the fulminant systemic course favored a clinical diagnosis of ANKL over extranodal NK/T-cell lymphoma. Despite chemotherapy, the patient died 24 days after admission. ANKL can mimic orbital cellulitis or idiopathic orbital inflammation, even without nasal involvement. Refractory orbital inflammation should prompt early biopsy and hematological evaluation to exclude NK/T-cell neoplasms.

## Introduction

Natural killer/T-cell lymphoma (NKTL) is a group of malignancies arising from NK cells or cytotoxic T cells, most of which are associated with Epstein-Barr virus (EBV). It is rare, comprising only 1%-3% of orbital non-Hodgkin lymphomas, and carries the highest lymphoma-related mortality among all ocular adnexal lymphoma subtypes [1,2]. In a large single-institution series of ocular adnexal NKTL, Lee et al. reported that 50% of patients presenting with primary ophthalmic symptoms had symptoms mimicking orbital cellulitis, with a mean symptom duration of 1.8 months before diagnosis [1]. Yang et al. similarly found that NK/T-cell lymphoma accounted for the largest proportion of misdiagnosed cases of secondary ocular adnexal lymphoma [2].

Aggressive natural killer cell leukemia (ANKL) is the most fulminant NK-cell neoplasm and accounts for less than 1% of all non-Hodgkin lymphomas. It predominantly affects young to middle-aged East Asians, is associated with EBV infection in approximately 90% of cases, and has historically had a median overall survival of less than two months [3]. In a recent Japanese nationwide cohort, median overall survival was 5.5 months, with improved outcomes after L-asparaginase-containing chemotherapy followed by allogeneic hematopoietic stem cell transplantation [4]. Extranodal NK/T-cell lymphoma (ENKL) and ANKL are EBV-associated cytotoxic NK-cell neoplasms with an overlapping immunophenotype (CD56+/EBV-encoded small RNA (EBER)+/cytotoxic). Still, they are distinct entities with different therapeutic implications: ENKL is a mass-forming extranodal lymphoma typically centered on the nasal cavity, whereas ANKL is a leukemic disease characterized by systemic proliferation of neoplastic NK cells involving the bone marrow, peripheral blood, liver, and spleen and has a far more fulminant course [3,4]. Because sinonasal involvement, classically regarded as a hallmark of ENKL, may be absent in primary orbital disease [1,5], neither the site of presentation nor the immunophenotype alone can reliably distinguish the two, and early tissue diagnosis is essential.

In the orbit, these neoplasms enter a differential diagnosis dominated by infectious orbital cellulitis and idiopathic orbital inflammation (IOI), a diagnosis of exclusion characterized by non-specific inflammation that typically responds to corticosteroids. Both mimics may delay recognition of malignancy. We report a case of ANKL presenting as orbital cellulitis without nasal involvement, with transient corticosteroid responsiveness that mimicked IOI, followed by rapid systemic deterioration and death within 24 days of admission.

## Case presentation

Outpatient course

A 50-year-old Japanese man with a history of right maxillary sinus opacity noted one year earlier and unexplained fever with hepatic dysfunction four months before referral presented with a two-month history of progressive right eyelid swelling. He had failed topical therapy at a local clinic. Acute worsening six days before referral prompted emergency transfer to a nearby hospital, where sinusitis was excluded by otolaryngology consultation, and intravenous ceftriaxone was given for two days under a diagnosis of orbital cellulitis without improvement. He was then referred to our institution.

Best-corrected visual acuity (BCVA) was 0.5 in the right eye and 1.2 in the left eye. Marked right eyelid swelling, conjunctival chemosis, and proptosis were noted, with restriction of ocular motility in all directions, most pronounced in downgaze (Figure 1).

**Figure 1:**
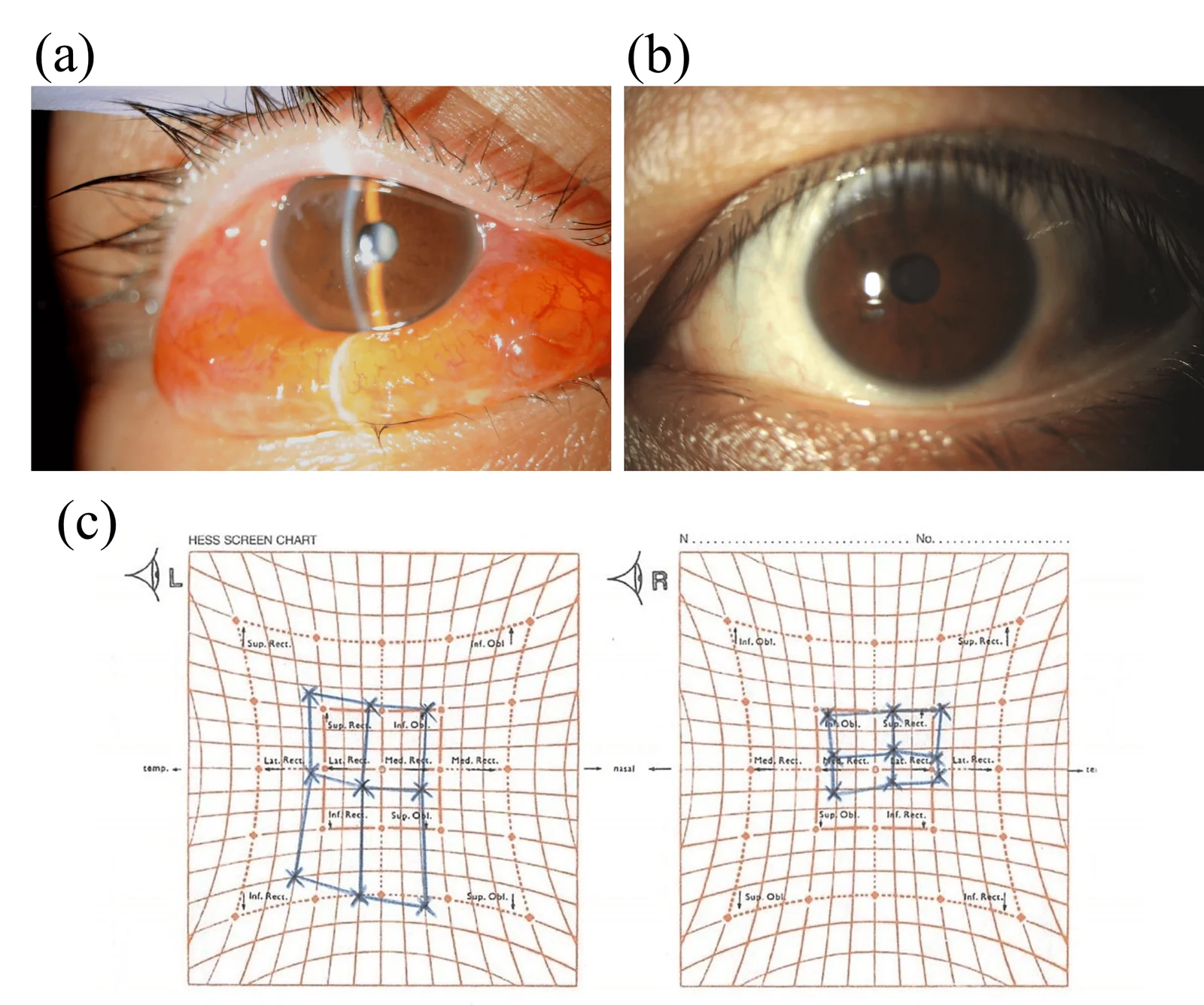
Slit-lamp photographs and ocular motility assessment at initial presentation (a) The right eye shows marked conjunctival chemosis and hyperemia. (b) The left eye shows a normal appearance for comparison. (c) Hess screen chart demonstrating restriction of right eye motility in all directions, with the most pronounced limitation in downgaze, corresponding to enlargement of the right inferior rectus on MRI (Figure 2).

Relative afferent pupillary defect was absent; slit-lamp and fundus examinations were unremarkable. MRI showed diffuse heterogeneous short TI inversion recovery hyperintensity in the right orbital fat, inferior rectus enlargement to its tendinous insertion, and gadolinium enhancement of orbital and periorbital tissues (Figure 2). The right maxillary sinus showed a pre-existing opacity consistent with a retention cyst, without mucosal thickening, bony destruction, or continuity with the orbital lesion, arguing against a sinogenic etiology.

**Figure 2:**
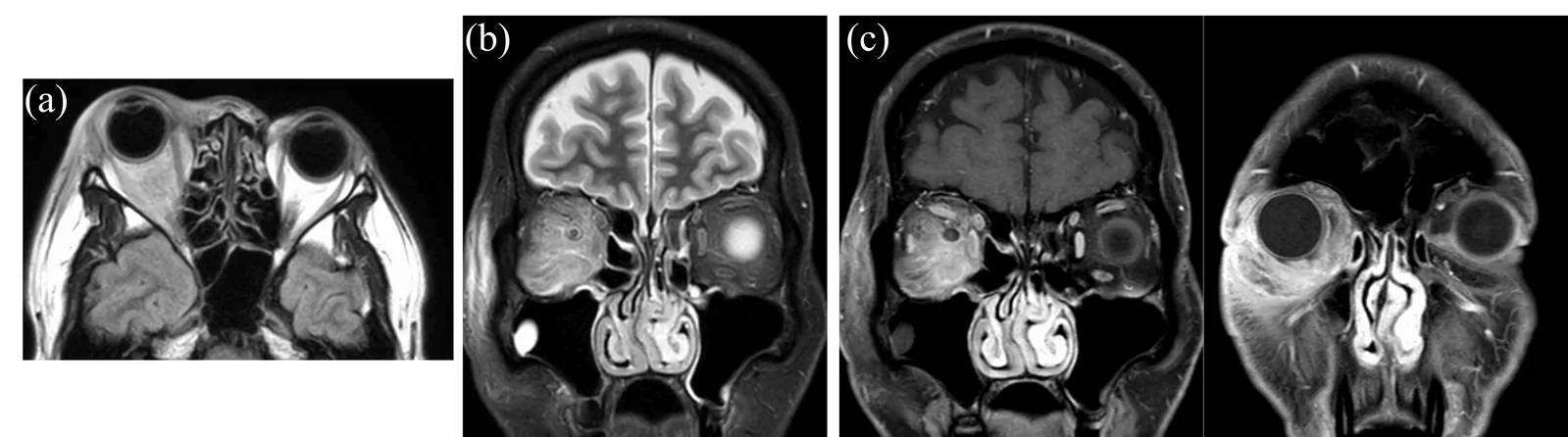
MRI at initial presentation (a) FLAIR axial sequence showing right orbital proptosis. (b) STIR coronal sequence demonstrating diffuse heterogeneous hyperintensity with poorly defined borders within the right orbital fat; a pre-existing right maxillary sinus opacity without bony destruction or orbital continuity is noted. (c) Gadolinium-enhanced T1-weighted coronal images at two different levels, showing enhancement of the right orbital and periorbital soft tissues. FLAIR, fluid-attenuated inversion recovery; STIR, short TI inversion recovery.

Laboratory evaluation revealed elevated hepatobiliary enzymes (aspartate aminotransferase (AST): 146 U/L, alanine aminotransferase (ALT): 273 U/L, γ-glutamyl transpeptidase: 672 U/L; total bilirubin: 2.5 mg/dL) and EBV serology consistent with past infection. The complete blood count (CBC), lactate dehydrogenase (LDH), soluble interleukin-2 receptor (sIL-2R), IgG4, and antineutrophil cytoplasmic antibody were within normal limits (Table 1), as were thyroid function tests, and C-reactive protein was only minimally elevated (0.32 mg/dL). Quantitative plasma EBV-DNA testing was not performed at this stage because IOI was the leading differential diagnosis.

Oral prednisolone (PSL) 40 mg/day with levofloxacin was initiated. Eyelid swelling markedly improved at eight-day follow-up (Figure 3b); however, symptoms relapsed at 14 days with declining BCVA and recurrent chemosis (Figure 3c). Antibiotic adjustment and PSL tapering failed to prevent progression. Nineteen days after the first visit, the patient presented with worsening proptosis, ocular pain, fever, and nausea and was admitted for intravenous methylprednisolone pulse therapy (1,000 mg/day × 3 days).

**Figure 3:**
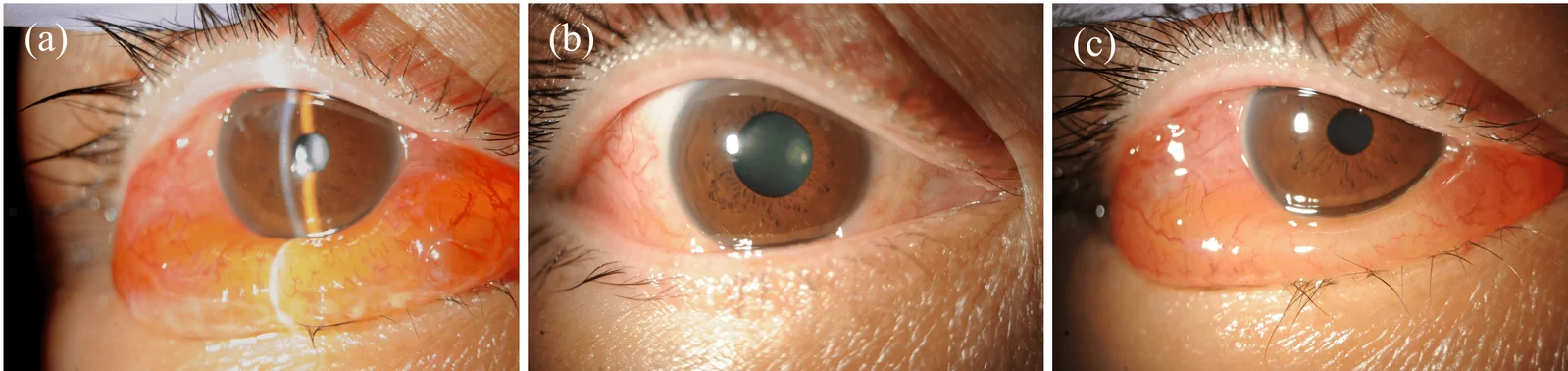
Slit-lamp photographs illustrating the corticosteroid response (a) At initial presentation (the same photograph as Figure 1a, shown for comparison of the clinical course): marked conjunctival chemosis and hyperemia. (b) Day 8 of prednisolone 40 mg/day: marked improvement. (c) Day 14: relapse of chemosis and hyperemia with declining visual acuity.

Inpatient course and diagnosis

Orbital swelling initially improved after pulse therapy, but the patient lost light perception in the right eye on day 3. Fundus fluorescein angiography was performed to exclude a retinal or choroidal vaso-occlusive process, such as central retinal artery occlusion or ocular ischemic syndrome. Retinal perfusion was preserved without choroidal filling delay, and the study was otherwise unremarkable, arguing against an intraocular vascular occlusive event. Over subsequent days, progressive thrombocytopenia (28 × 10³/µL on day 7), worsening hepatic dysfunction (AST: 692 U/L, ALT: 763 U/L by day 7), persistent fever, and bilateral pulmonary infiltrates developed. On day 9, sIL-2R rose to 13,605 U/mL, ferritin to 38,640 ng/mL, and fibrin/fibrinogen degradation products and D-dimer were markedly elevated. Quantitative plasma EBV-DNA was markedly elevated at 7.49 Log IU/mL, raising suspicion for lymphoma-associated hemophagocytic lymphohistiocytosis (HLH; also termed hemophagocytic syndrome) (Table 1). Automated differential counts reported reactive lymphocytes at 1.0% on day 8; a dedicated morphological search for neoplastic large granular lymphocytes was not undertaken, and no increase in atypical lymphoid cells was documented on peripheral blood examination.

**Table 1:** Laboratory findings at initial presentation and during the inpatient course —, not measured at that time point. *At the first visit to our institution. At this stage, the complete blood count, LDH, and soluble IL-2 receptor were within normal limits, and CRP was only minimally elevated, with isolated elevation of hepatobiliary enzymes and EBV serology indicating past infection. †Days 7-9 of admission, when progressive thrombocytopenia, worsening hepatic dysfunction, and markedly elevated ferritin, soluble IL-2 receptor, FDP/D-dimer, and plasma EBV-DNA raised suspicion for hemophagocytic lymphohistiocytosis. Complete blood count and hepatobiliary enzymes were obtained on day 7; ferritin, soluble IL-2 receptor, FDP/D-dimer, CRP, and plasma EBV-DNA were obtained on day 9. Plasma EBV-DNA was quantified by real-time PCR and is expressed as Log IU/mL. Values in bold denote the diagnostically decisive abnormalities. Reference ranges are those of our institution's clinical laboratory; values may vary by assay platform. ALP, alkaline phosphatase; ALT, alanine aminotransferase; ANCA, antineutrophil cytoplasmic antibody; AST, aspartate aminotransferase; BUN, blood urea nitrogen; CRP, C-reactive protein; EBNA, Epstein-Barr nuclear antigen; EBV-DNA, Epstein-Barr virus DNA; ESR, erythrocyte sedimentation rate; FDP, fibrin/fibrinogen degradation product; IFCC, International Federation of Clinical Chemistry and Laboratory Medicine; LDH, lactate dehydrogenase; MCH, mean corpuscular hemoglobin; MCHC, mean corpuscular hemoglobin concentration; MCV, mean corpuscular volume; MPO, myeloperoxidase; VCA, viral capsid antigen; γ-GTP, γ-glutamyl transpeptidase.

Parameter	At initial presentation^*^	Days 7-9 (deterioration)†	Reference range	Unit
Complete blood count				
White blood cells	5,700	7,100	3,300-8,600	/µL
Red blood cells	5.05 × 10⁶	5.17 × 10⁶	4.35-5.55 × 10⁶	/µL
Hemoglobin	14.6	14.9	13.7-16.8	g/dL
Hematocrit	44.5	—	40.7-50.1	%
MCV	88.1	—	83.6-98.2	fL
MCH	28.9	—	27.5-33.2	pg
MCHC	32.8	—	31.7-35.3	g/dL
Platelets	203 × 10³	28 × 10³	158-348 × 10³	/µL
Biochemistry				
Total protein	7.7	—	6.6-8.1	g/dL
Albumin	4.3	—	4.1-5.1	g/dL
AST	146	692	13-30	U/L
ALT	273	763	10-42	U/L
LDH	211	—	124-222	U/L
ALP (IFCC)	198	207	38-113	U/L
γ-GTP	672	576	13-64	U/L
Total bilirubin	2.5	—	0.4-1.5	mg/dL
BUN	8	—	8-20	mg/dL
Creatinine	0.68	—	0.65-1.07	mg/dL
Glucose	103	—	73-109	mg/dL
Sodium	140	—	138-145	mmol/L
Potassium	4.2	—	3.6-4.8	mmol/L
Chloride	107	—	101-108	mmol/L
CRP	0.32	4.89	≤0.14	mg/dL
ESR	2	—	2-10	mm/h
Coagulation and hemophagocytic markers				
FDP	—	28.6	<5.0	µg/mL
D-dimer	—	17.3	<1.0	µg/mL
Ferritin	—	38,640	30-400	ng/mL
Soluble IL-2 receptor	390	13,605	121-613	U/mL
Immunoserology				
IgG	1,459	—	861-1,747	mg/dL
IgA	365	—	93-393	mg/dL
IgM	78	—	33-183	mg/dL
IgG4	34.6	—	4.5-117	mg/dL
Complement C3	165	—	73-138	mg/dL
Complement C4	39	—	11-31	mg/dL
CH50	72.1	—	30-45	U/mL
Rheumatoid factor	3	—	<15	IU/mL
Antinuclear antibody	Negative	—	<1:40	titer
PR3-ANCA	<1.0	—	<3.5	IU/mL
MPO-ANCA	<1.0	—	<3.5	IU/mL
Epstein-Barr virus				
Plasma EBV-DNA	—	7.49	Not detected	Log IU/mL
VCA-IgG	80	—	<10	titer
VCA-IgM	<10	—	<10	titer
EBNA	40	—	<10	titer

Flow cytometric immunophenotyping of the bone marrow aspirate showed CD2+, CD7+, CD56+, and HLA-DR+ cells (pan-HLA-DR antibody; subgroup not determined), without CD13 or CD34. Histopathological examination of the bone marrow biopsy, performed on day 9, revealed infiltration of the intertrabecular spaces by medium-sized lymphoid cells. Hemophagocytosis was occasionally observed (Figure 4a). Immunohistochemically, the neoplastic cells were positive for CD3 (cytoplasmic), CD7, and CD56 (Figure 4b) but negative for CD10, CD20, CD30, CD34, and CD117. EBER in situ hybridization was positive in the neoplastic cells (Figure 4c). Liver biopsy revealed infiltration of the portal areas and hepatic sinusoids by medium-sized neoplastic cells, accompanied by nuclear debris (Figure 4d). The neoplastic cells exhibited an immunophenotype identical to that observed in the bone marrow and were also positive for EBER by in situ hybridization. In addition, the neoplastic cells in the liver were positive for T-cell intracellular antigen-1 (TIA-1) and granzyme B.

**Figure 4:**
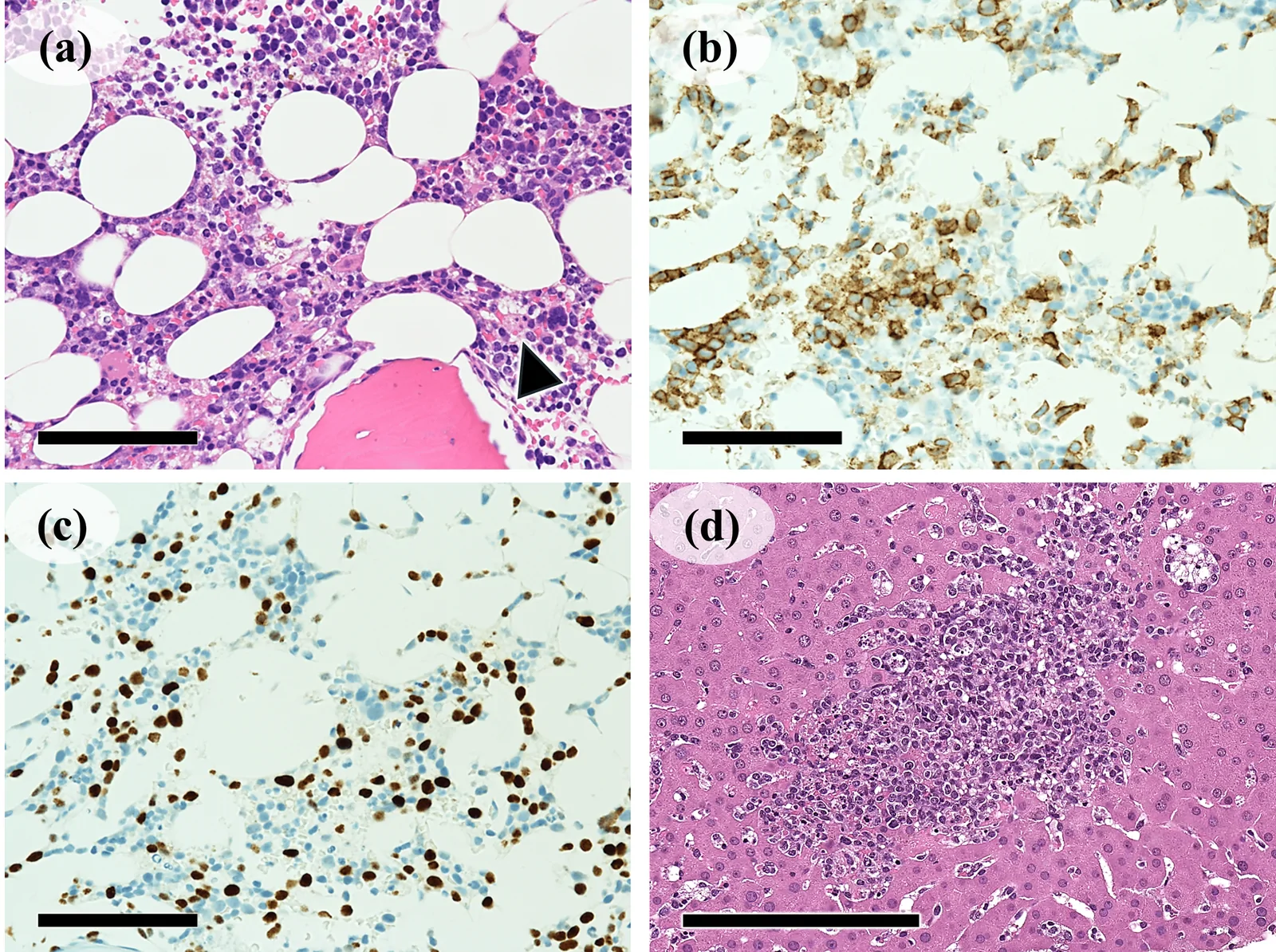
Histopathological findings (a-c) Bone marrow biopsy. Medium-sized lymphoid cells infiltrate the intertrabecular spaces (a), with hemophagocytosis also observed (arrowhead). The neoplastic cells show CD56 expression by immunohistochemistry (b) and EBER positivity by in situ hybridization (c). (d) Liver biopsy. Similar neoplastic cells also infiltrate the portal areas and hepatic sinusoids, without mass formation (a, hematoxylin and eosin, scale bar = 100 µm; b, CD56, scale bar = 100 µm; c, EBER in situ hybridization, scale bar = 100 µm; d, hematoxylin and eosin, scale bar = 200 µm). EBER, EBV-encoded small RNA.

Taken together, the evidence supporting ANKL comprised leukemic bone marrow infiltration by EBER+/CD56+ NK cells, concurrent sinusoidal and portal infiltration of the liver without a discrete hepatic mass by the same cell population, in which expression of the cytotoxic proteins TIA-1 and granzyme B was also demonstrated, absence of nasal or sinonasal involvement on imaging, and a fulminant clinical course complicated by features of HLH. Flow cytometric quantification of circulating neoplastic NK cells in peripheral blood was not performed. ANKL was diagnosed based on bone marrow and liver findings together with the clinical course, and the patient was transferred to hematology.

Outcome

Cyclophosphamide, doxorubicin, vincristine, and PSL (CHOP) chemotherapy was initiated on day 10. However, rapidly deteriorating performance status and active disseminated intravascular coagulation (DIC) precluded use of an L-asparaginase-containing regimen such as dexamethasone, methotrexate, ifosfamide, L-asparaginase, and etoposide (SMILE). DIC progressed and proved refractory to transfusion support. Enterococcus faecium bacteremia developed on day 14. A hemorrhagic stroke on day 23 resulted in death on day 24 after admission. The clinical course is summarized in Figure 5.

**Figure 5:**
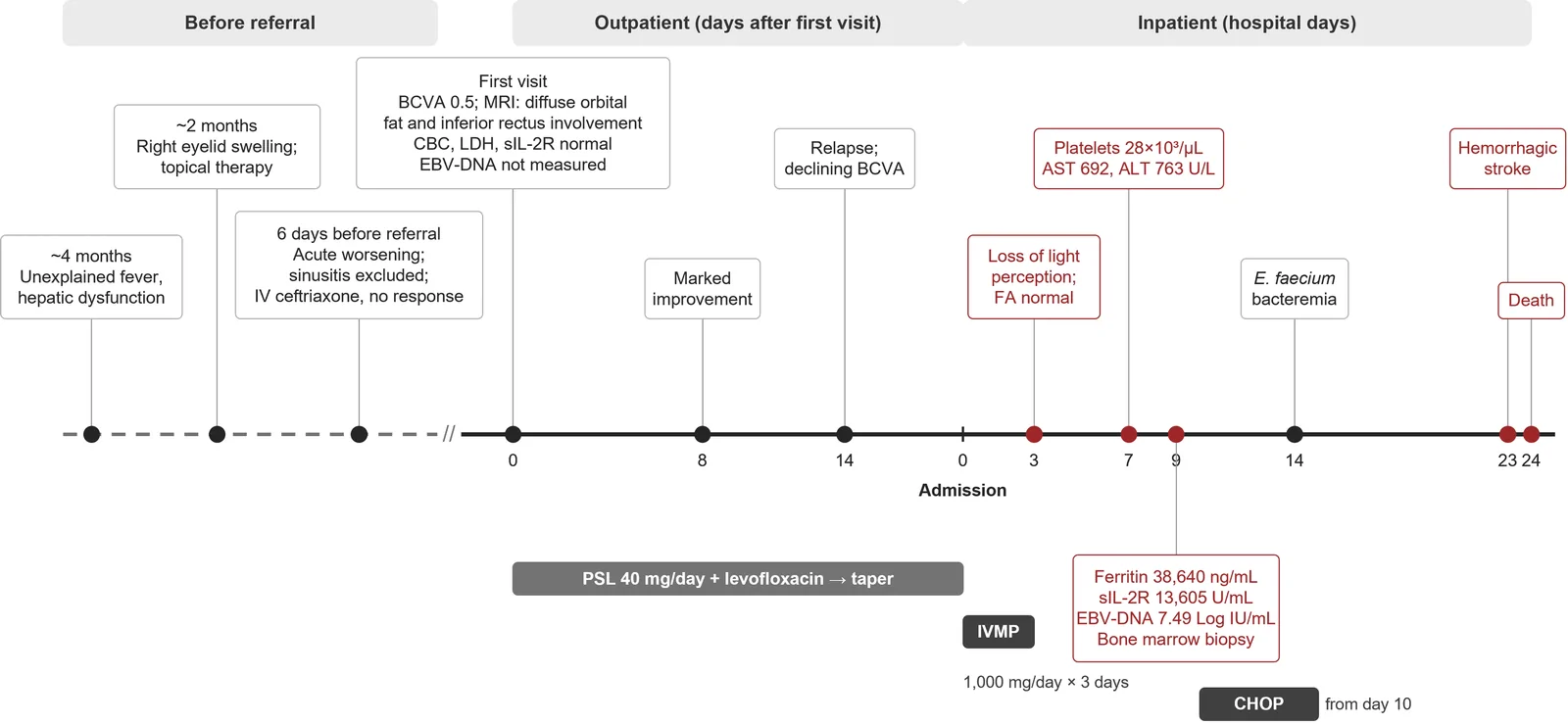
Timeline of the clinical course Events before referral are not drawn to scale. The outpatient and inpatient periods are shown on a continuous scale; hospital day 0 corresponds to day 19 after the first visit. Red markers indicate the principal events of the systemic deterioration. ALT, alanine aminotransferase; AST, aspartate aminotransferase; BCVA, best-corrected visual acuity; CBC, complete blood count; CHOP, cyclophosphamide, doxorubicin, vincristine, and prednisolone; EBV, Epstein-Barr virus; FA, fluorescein angiography; IVMP, intravenous methylprednisolone; LDH, lactate dehydrogenase; MRI, magnetic resonance imaging; PSL, prednisolone; sIL-2R, soluble interleukin-2 receptor.

## Discussion

NK/T-cell neoplasms are rare but are increasingly recognized as mimics of orbital inflammatory disease [6]. In a series of 11 patients with orbital NK/T-cell neoplasms, all were initially misdiagnosed as inflammatory pseudotumor, orbital cellulitis, or conjunctivitis, and five of the eight patients treated with corticosteroids or antibiotics before referral experienced transient symptom relief followed by recurrence. In one patient, as in ours, bone marrow involvement and hemophagocytic syndrome emerged during treatment, and ANKL was diagnosed [7]. In a recent report of orbital ENKL simulating orbital inflammatory disease, the orbital lesion progressed despite intravenous methylprednisolone [8], whereas our patient showed a transient corticosteroid response. To our knowledge, ANKL presenting as antibiotic-refractory, cellulitis-like orbital inflammation with transient corticosteroid responsiveness and normal blood counts at presentation has not previously been described in comparable detail.

Several factors made the diagnosis difficult in our patient: (i) the paucity of hematological abnormalities at presentation; (ii) transient responsiveness to corticosteroids; (iii) the absence of sinonasal involvement; and (iv) the barriers to early orbital biopsy. First, CBC, LDH, and sIL-2R were normal at the first visit, and EBV serology indicated only past infection. This pattern may reflect an early stage of ANKL, in which neoplastic NK cells are too few to cause hematological abnormalities. The mild hepatic dysfunction noted at the first visit may have reflected early hepatic involvement, but other causes could not be excluded, which made liver biopsy difficult to justify at that point. Quantitative plasma EBV-DNA testing, which may detect subclinical EBV-associated lymphoproliferation before hematological markers become abnormal, was not performed at the first visit because the clinical findings pointed primarily to IOI. When it was obtained on day 9 of admission, the plasma EBV-DNA level was markedly elevated at 7.49 Log IU/mL; earlier testing might have provided the clue that led to the diagnosis of an NK-cell neoplasm.

Second, transient corticosteroid responsiveness delayed recognition of the underlying neoplasm. As in our patient, Lee et al. reported a case in which orbital swelling resolved with oral corticosteroids, recurred after cessation, and improved only partially with intravenous corticosteroids; biopsy then revealed NK/T-cell lymphoma with bone marrow and liver involvement [1]. In our patient, eyelid swelling improved after pulse corticosteroid therapy, yet light perception in the right eye was lost on day 3 of admission. Fluorescein angiography excluded an intraocular vaso-occlusive event and localized the responsible lesion posterior to the globe. We presume that tumor infiltration and the angiodestructive behavior of NK/T-cell neoplasms at the orbital apex caused compressive and ischemic optic neuropathy, which corticosteroids could not halt even though they reduced superficial edema.

Third, the absence of sinonasal involvement on imaging argued against an NK/T-cell neoplasm. Although orbital NK/T-cell neoplasm most commonly arises by direct extension from the nasal cavity or paranasal sinuses, primary orbital disease without sinonasal involvement has been documented in a minority of cases [1,5,9]; in the series of Li et al., 4 of 11 patients (36.4%) had orbital disease without nasal involvement [7]. In our patient, the pre-existing right maxillary sinus opacity, present on imaging from one year prior and lacking bony destruction or orbital continuity, was a retention cyst unrelated to the orbital lesion, as supported by otolaryngological evaluation at the referring hospital that excluded active sinusitis.

Fourth, early orbital biopsy was difficult to pursue. Li et al. recommended early biopsy when anti-inflammatory and corticosteroid therapies are ineffective [7], but orbital biopsy was never performed in our patient: IOI was the leading diagnosis at the first visit, and the initial steroid response appeared to confirm it. When the inflammation relapsed, the general condition was already deteriorating, and thrombocytopenia and DIC made an invasive orbital procedure hazardous; bone marrow and liver biopsies were therefore preferred as less invasive sources of tissue. In retrospect, the relapse after an initial steroid response was the point at which biopsy, together with measurement of plasma EBV-DNA, should have been performed because the window in which orbital biopsy is both informative and safely feasible closes as the disease becomes systemic. Diffuse inflammation without a discrete mass may discourage biopsy, but orbital fat and an enlarged extraocular muscle can both be sampled, and orbital tissue is diagnostic even when the sinonasal cavities are spared [10]. Sampling should be generous and may need to be repeated because angioinvasion and necrosis often render biopsies inconclusive [6]; moreover, in ANKL, orbital findings may reflect direct NK-cell infiltration [11] but may also be reactive [12].

The distinction between ANKL and ENKL is clinically important because the two share an overlapping immunophenotype but differ in treatment implications [3,13]. According to the fifth edition of the WHO classification of haematolymphoid tumors, ANKL overlaps clinicopathologically with disseminated ENKL and is distinguished from it chiefly by a fulminant systemic onset and the absence of nasal disease [14]; our patient showed both features. The leukemic pattern of bone marrow infiltration and the concurrent sinusoidal and portal infiltration of the liver without a mass lesion further favored ANKL [13]. Although flow cytometric quantification of circulating neoplastic NK cells and a dedicated morphological search of the peripheral blood smear were not performed, peripheral blood involvement is not among the features that distinguish the two entities. In a recent nationwide cohort of ANKL, SMILE was associated with a higher overall response rate than CHOP-like therapy (66% vs. 31%) and with better overall survival [4]; however, SMILE could not be administered in this case because of the patient's rapidly deteriorating status and fulminant DIC at the time of diagnosis.

For ophthalmologists, the key lesson is that NK/T-cell neoplasms should be considered when orbital inflammation is refractory to antibiotics or relapses after an initial corticosteroid response, even without sinonasal involvement. Systemic warning signs are easily overlooked: in our patient, unexplained fever and hepatic dysfunction preceded the orbital symptoms, and fever, progressive thrombocytopenia, and marked elevation of ferritin, sIL-2R, and plasma EBV-DNA accompanied the subsequent deterioration. Because these markers may be normal or unmeasured at presentation, they should be reassessed when orbital inflammation does not resolve. Abnormal results, or failure of the orbital lesion to respond durably, should prompt urgent orbital biopsy with CD56 immunostaining, EBER in situ hybridization, and cytotoxic markers (TIA-1 and granzyme B), together with bone marrow examination.

## Conclusions

ANKL may present with orbital inflammation that mimics cellulitis or IOI, even without nasal involvement. In our patient, although unexplained fever and hepatic dysfunction had preceded the orbital symptoms, the orbital findings were the first manifestation to prompt specialist evaluation, placing the ophthalmologist in the first position to suspect this rapidly fatal systemic malignancy. Transient corticosteroid responsiveness can further delay diagnosis. Diagnosis rests on demonstrating an EBER-positive cytotoxic NK-cell neoplasm in involved tissue. The acute, fulminant systemic course and the absence of nasal disease matched the features by which the current WHO classification distinguishes ANKL from disseminated ENKL; leukemic bone marrow infiltration and sinusoidal infiltration of the liver without a mass lesion further supported this diagnosis. Refractory orbital inflammation warrants early biopsy and hematologic evaluation, including sIL-2R, ferritin, and plasma EBV-DNA.
